# Fuzzy MCDM models for selection of the tourism development site: the case of Azerbaijan

**DOI:** 10.12688/f1000research.109709.1

**Published:** 2022-03-14

**Authors:** Aziz M. Nuriyev

**Affiliations:** 1 Azerbaijan State Oil and Industry University, Khazar University, Baku, Azerbaijan

**Keywords:** Z-TOPSIS, Z-PROMETHEE, Z-number, Direct Calculations with Z-numbers, Normalization, Swing Weights, Tourism Site selection

## Abstract

**Background: **One of the vital issues in promoting the sustainable tourism industry in developing countries, including Azerbaijan, is the well-grounded selection of tourism sites. Applying traditional approaches as a solution to this task, does not provide a relevant result in all cases in these countries due to local specifics of the tourism, the incompleteness of statistical data, the high-level uncertainty of the internal and external environment, and the questionable reliability of the available information.

**Methods: **Since the statistical data are limited, and conventional formalization tools used for uncertainty description do not consider the reliability degree of the data, it is suggested to make decisions based on the Z-extension of fuzzy logic. A Delphi panel with the expert group is conducted to obtain the information required for the model development. Fuzzy Z-information-based TOPSIS and PROMETHEE methods are applied for the problem solution. Within these approaches Z-number-based procedures of the decision matrix normalization, defining the distance between solutions and the preference function, and swing weights determination are realized. Direct computations with Z-numbers are implemented.

**Results**: By applying Z-number-based multi-criteria decision-making methods, five potential regions of Azerbaijan have been evaluated for six criteria. The criteria reflect government policy to the development of the regions, economical, geographical, environmental factors, and infrastructure of the locations. Derived solutions are comparable in sense of sites ranking, and similar results were obtained using both methods. Direct calculations allow obtaining results based on the linguistic Z-evaluations of experts without distorting transformations.

**Conclusion**: The managerial decision-making problems in the tourism sector, raised due to the aforementioned barriers, can be successfully resolved by applying Z-number-based multi-criteria approaches. The obtained results allow increasing a range of the decision-making tasks under a high degree of uncertainty to be solved for sustainable development studies and other areas.

## Introduction

Tourism site selection is a very important issue for the sustainable development of the tourism industry in developing countries. Even though there are many tools for auditing the potential locations, the development of tourism in such countries, including Azerbaijan, has its own specifics. Simple copying of the method that was successful in one application, does not provide a relevant result in all identical cases. Moreover, the paradigm of sustainable tourism development requires a flexible combination of the scarcely compatible requirements such as maximizing socio-economic benefits for the environment, society, visitors, cultural heritage sites and minimizing negative impacts.

Analysis of the literature shows that there is a time lag between tourism site location studies carried out in developed and developing countries. Developed countries, based on their economic power, studied and implemented the research results earlier. At present, in these countries, almost all potential areas for tourism development have been already used. In developing countries, such studies began much later. As economies are growing, these countries are looking for development of the tourism and recreational areas. The development of tourism stimulated the hospitality industry-related studies in developing countries, including Azerbaijan. The main studies devoted to tourism site location refer to the countries of Asia and Africa. Lack of well-established approaches to tourism development site selection in the tourism market of developing countries causes great interest in such studies for researchers from these countries.

Multi-criteria decision-making (MCDM) approaches that allow considering together a set of alternatives, factors, and contradictory criteria are widely used for site location selection ^1^ for energy generation, logistics, public services, and retail facilities. The tourism sector and hospitality industry are also successfully using multi-criteria analysis for managerial decision-making. ^2^ The selection of the location for the construction of the tourism and hospitality objects is one of the complex managerial multi-criteria tasks that has become increasingly important in recent years in developing countries. It is directly related to such factors as the growth of tourism, the high competition, the necessity for new approaches to the creation of hospitality facilities, and the increasing sophistication of the tourism development location selection decisions.

A two-stage procedure for the solution of the resort location selection was performed by Juan & Lin in 2013. ^3^ Selection criteria were identified by modified Delphi and then evaluated by Analytical Hierarchy Process (AHP). The ecotourism site selection task was also realized by applying Delphi and AHP methods. ^4^ The use of the AHP method for the selection of the tourism sites were presented by Abed *et al*., in 2011 and by Hadiwijaya *et al*., in 2018. ^5^^,^^6^ Analytic network process (ANP) method was applied by Bunruamkaew and Murayama in 2011 for the problem solution. ^7^ Strengths, weaknesses, threats, opportunities (SWOT) technique of strategic analysis was used for site selection for tourism villages. ^8^ The spiritual tourism destination selection topic in India was studied by Satpathy & Mahalik in 2010 and AHP was used. ^9^ For the selection of the tourism facility location for future investment TOPSIS (Technique for Order of Preference by Similarity to Ideal Solution), VIKOR (VIseKriterijumska Optimizacija I Kompromisno Resenje), AHP methods were used. ^10^ Fuzzy MCDM approach - Fuzzy DEMATEL (Decision Making Trial and Evaluation) was used for ecotourism site selection. ^11^ ANP and TOPSIS were used for selection of the optimal tourism site in the Integrated Coastal Zone Management in a fuzzy environment. ^12^ AHP and TOPSIS were used for the location selection of new campus for hospitality and hotelier majors. ^13^ MCDM methods are also widely used for hotel location selection. ^14^ MCDM methods such as the Best-Worst Method (BWM) and Weighted Aggregated Sum Product Assessment (WASPAS), ^15^ adapted Stepwise Weight Assessment Ratio Analysis (SWARA) and Weighted Sum Preferred Level of Performances (WS PLP), ^16^ Pivot Pairwise Relative Criteria Importance Assessment (PIPRECIA) and Additive Ratio Assessment (ARAS), ^17^ as well as fuzzy methodologies Fuzzy AHP (FAHP) ^18^ and Fuzzy TOPSIS (FTOPSIS) ^19^ were implemented for hotel location selection.

It is necessary to underline that during decision-making in the tourism sector of developed countries, especially for further activities, the managers often deal with information that should be characterized as imperfect. The structural thesaurus given in ^20^ reflects aspects of imperfect information such as imprecision, inconsistency, and uncertainty. In tourism, there are often arising situations associated with inaccuracy, incompleteness, or partial reliability of the information available for decision-making. These specifics are generated by the involvement of various actors in this sphere, and the impossibility to describe all processes and components by the perfect information.

In the case of developing countries, the level of uncertainty increases even more because the statistical apparatus for such territories is not fully formed and its saturation with information differs from traditional destinations. This is due to various factors - the lack of tradition of conducting surveys, the reluctant participation of the population, the limited number of trained personnel, and financial difficulties. However, the absence of such statistics does not obviate the need to make decisions. In addition, in some cases, through applying statistical analysis, it is difficult to find out the dependency between tourist flows and infrastructural or socio-economic factors. ^21^ The need to make decisions based on the available data without adequate statistical history, as well as the combination of qualitative parameters with quantitative information, necessitate the involvement of experts to obtain aggregate estimates. Aggregated linguistic assessments allow decision-makers to get a more adequate idea of the tasks being solved, especially at the initial stage or meta-level. For example, the *distance* to the object can be estimated by many people on the map. However, aggregated expert assessment of *accessibility*, including distance, road quality, traffic density, accident rate, the presence of carriers, etc., makes it possible to assess the situation more adequately.

To solve the abovementioned problems, the usage of the concept of Z-numbers ^22^ is proposed, which allows describing imperfect information in expressions that are as close as possible to the natural language, used by participating persons. Analysis of tourism shows that there are many examples of the use of information that can be best described by Z-numbers. For example, *Tourism flow in the next quarter* = ( *decrease, probably*) and *Health care status* = ( *high, very likely*). Since its introduction, the Z-number paradigm has attracted an increasing number of researchers. Currently, many works related both to theoretical aspects of the Z-numbers and their application in various fields have been published. ^23^^–^^28^ However, despite this, the number of papers devoted to the application of the approaches, based on direct calculations with Z-numbers in decision-making tasks, is still relatively small. Many researchers prefer to convert Z-numbers during calculations. In this case, the initial information with a high degree of uncertainty has been transformed into a formalism with less uncertainty - fuzzy or crisp numbers.

In this paper, the solution for tourism site location in Azerbaijan is put forward. The approach is based on the utilization of Z-numbers in a multi-criteria decision-making model. The proposed approach allows decision-making based on expert assessments expressed by Z-information. The calculation mechanism and tools, allowing direct calculations without losing the initial information contained in the Z-numbers, are presented.

The purpose of this research is selection tourism development site by application of Z-number-based fuzzy MCDM models. The applicability of Z-numbers, describing the uncertain information, in multi-criteria decision-making are studied. Z-extensions of the TOPSIS and PROMETHEE (Preference Ranking Organization METHod for Enrichment of Evaluations) methods, based on direct calculations with Z-numbers, are considered. Decision matrix normalization and criteria weighting techniques for Z-number-based extensions are suggested.

The paper is organized as follows: Section Two presents Z-numbers and arithmetical operations technique with Z-numbers, direct calculations with Z-number-based TOPSIS and PROMETHEE methods, approach to normalization of the decision matrix and determination weights of criteria. Section Three presents potential regions of Azerbaijan to be studied for the tourism site location selection, criteria of selection, and results of calculations according to the Z-TOPSIS and Z-PROMETHEE approaches. Section Four presents the discussion and Section Five presents the conclusions.

## Methods

### Ethics

Confidentially was assured to all participating experts and informed oral consent was also obtained.

***Operations with Z-numbers***

Definition One. *Z-number.* ^22^

Continuous or discrete Z-number is an ordered pair (A, B) of fuzzy numbers. Part A, expressed by continuous/discrete fuzzy number (FN), is a restriction on the values of uncertain variable X. Part B, expressed by continuous/discrete fuzzy number, is a measure of the reliability or certainty of A. B _1_ and B _2_ are sets of probability measures of A _1_ and A _2_, respectively.

Example: *X* is *A* with certainty degree *B* or *demand* is ( *high, very sure*)

If we express the same values in the form of trapezoidal or triangular membership functions (MF), then Z-numbers can be represented as in the following example:

$$\left(10,15,20,25\right)\;\left(0.4,0.6,0.8,1\right)\;\text{or}\;\left(12.5,17.5,22.5\right)\;\left(0.5,0.7,0.9\right)$$

Definition Two. *Arithmetic operations on Z-numbers.* ^29^

If *Z _1_* and *Z _2_* are two Z-numbers with parts *A* and *B* expressed as ( *A _1_*, *B _1_*) and ( *A _2_*, *B _2_*), and * is one of the binary arithmetic operations (+, -, ^.^, /), then this operation on Z-numbers is defined by the formula

$$Z_{12}\left(A_{12},B_{12}\right)=\left(A_{1},B_{1}\right)*\left(A_{2},B_{2}\right)$$ (1)

Part *A* of *Z _12_* is computed under the rules of arithmetic operations on fuzzy numbers *A _12_* = *A _1_* * *A _2_*.

The calculation of part *B _12_* of the Z-number *Z _12_* is a more complex task since this part defines the degree of confidence, which is expressed in terms of the theory of probability. To calculate *B _12_*, the methods, based on the fundamental principles of operations on Z-numbers, proposed by Zadeh in 2011, are used. ^22^ Calculation procedures were elaborated in details by Aliev in 2015-2017. ^29^^,^^30^ Based on the supp of *B _1_* and *B _2_*, expressing the fuzzy measures of parts *A _1_* and *A _2_*, the corresponding probability distributions are induced, the convolution of which specifies the set of fuzzy measures *A _12_* ( *supp* of *B _12_*). Further, the membership function for *B _12_* is defined.

Definition Three. *Fuzzy Pareto optimality principle-based ranking of Z-numbers.* ^29^

Two Z-numbers are compared as multi-attribute alternatives by calculating the degrees of optimality *do*( *Z _1_*) and *do*( *Z _2_*). These degrees are determined based on the number of components for which one Z-number dominates over another Z-number. Calculation of *do *( *Z _i_*) is a multi-stage process. At the first stage, A parts are normalized. Then intermediate functions *n _best_* ( *Z _i_, Z _j_*) *, n _equal_* ( *Z _i_, Z _j_*) *,* and *n _worst_* ( *Z _i_, Z _j_*) that are estimating how much one Z-number is superior, equivalent, or less with respect to the components A and B are calculated. Then according to the following formula, function *d* ( *Z*_*i*_, *Z*_*j*_) is calculated

dZiZj=0,ifnbestZiZj≤1−0.5∙nequalZiZj2nbestZiZj+nbestZiZj−2nbestZiZj (2)

If *d *( *Z _i_,Z _j_*) = *1,* then *Z _i_* is Pareto-dominated over *Z _j_.* If *d *( *Z _i_,Z _j_*) = *0*, *Z _i_* is not Pareto-dominated over *Z _j_.* Based on the values of the function *d* the degree of optimality of *Z _j_* is calculated by the formula

$$\mathrm{do}\;\left(Z_{i}\right)=1-d\;\left(Z_{i},Z_{j}\right)$$ (3)

*do *( *Z _i_*) determines the degree to which one Z-number is over than another. By other words

$$Z_{i}>Z_{j},\text{if}\;\mathrm{do}\;\left(Z_{i}\right)>\mathrm{do}\;\left(Z_{j}\right)$$

$$Z_{i}<Z_{j},\text{if}\;\mathrm{do}\;\left(Z_{i}\right)<\mathrm{do}\;\left(Z_{j}\right)$$

$$Z_{i}=Z_{j},\text{if}\;\mathrm{do}\;\left(Z_{i}\right)=\mathrm{do}\;\left(Z_{j}\right)$$

Definition Four. *Distance between Z-numbers.*

According to the ^30^^–^^32^ the distance between two Z-numbers Z _1_ and Z _2_, whose parts expressed by trapezoidal fuzzy numbers *A _1_* = ( *a _11_,a _12_,a _13_,a _14_*), *B _1_* = ( *b _11_,b _12_,b _13_, _14_*), *A _2_* = ( *a _21_,a _22_,a _23_,a _24_*), *B _2_* = ( *b _21_,b _22_,b _23_, _24_*), are calculated according to the following formula

$$D\left(Z_{1},Z_{2}\right)=0.5∙\left\{\sum_{i=1}^{4}\left|a_{1i}-a_{2i}\right|+\sum_{j=1}^{4}\left|b_{1j}-b_{2j}\right|\right\}$$ (4)

***Z-extensions of the MCDM and direct calculations with Z-numbers***

Analysis of the recent research studies shows that for location selection tasks the following MCDM techniques are used: distance-based, pairwise comparison, outranking, and scoring methods. Therefore, for tourism development site selection two well-proven approaches from different groups of MCDM techniques, namely, distance-based method – TOPSIS and outranking method – PROMETHEE were chosen.

Since decision-makers and experts will use Z-information in the decision process, the methods used will be Z-TOPSIS and Z-PROMETHEE. Although there are publications in the literature on the use of Z-TOPSIS and Z-PROMETHEE, most of the implementations are oversimplifying method, when the Z-number is converted sequentially to a fuzzy number and then to a crisp number. A reasonable question arises – why to use Z-numbers if various conversions is carried out that distort the very essence of Z-information? It should be taken into consideration that in the case of transformations, the information contained in the Z-numbers will be partially lost.

In the approaches proposed in the paper, only at the final stages, to establish an order relation between the obtained values to select alternatives, crisp values of appropriate functions are used to define the dominance of one calculated Z-number over another or to determine the distance between Z-numbers.

The calculations with Z-numbers are based on the methodology specified in ^29^^–^^30^ and realized on Python-based SciPy software. This approach allows the solving of the optimization tasks required for the implementation of the arithmetic operations.

***Z-TOPSIS***

Applications of the classic and fuzzy TOPSIS are widely presented in research papers. The features of this method application in case of the use of Z-numbers and direct calculations with these numbers are outlined below.

Step 1. Defining a cost and benefit criteria.

Step 2 *.* Construction of the initial decision matrix ( *ZDMx*) with *m* rows (alternatives) and *n* columns (criteria). Each element of matrix is expressed by Z-number.

$$\mathrm{ZDMx}=\left[\begin{array}{cccc} z_{11} & z_{12} & \ldots & z_{1n}\\ z_{21} & z_{22} & \ldots & z_{2n}\\ \ldots & \ldots & & \ldots \\ z_{i1} & z_{i2} & \ldots & z_{\text{in}}\\ \ldots & \ldots & & \ldots \\ z_{m1} & z_{m2} & \ldots & z_{\mathrm{mn}} \end{array}\right]$$

Step 3. Normalization of the decision matrix.

There are various approaches by Lakshmi &Venkatesan in 2014 and Ploskas & Papathanasiou in 2019 for normalizing the decision matrix in TOPSIS, which have a common goal of bringing criteria in the comparable form and dimensionless quantities. ^33^^,^^34^ However, regardless of the approach, the order relation between alternatives for each criterion remains invariant. Taking these circumstances into account, for normalization of the decision matrix expressed by Z-numbers, the linear scale transformation ^35^ of part A is applied. If part A, for example, is expressed by triangular fuzzy number, then

$$A_{\mathrm{ij}}^{\mathrm{norm}}=\left(\frac{l_{\mathrm{ij}}}{r_{j}^{*}},\frac{m_{\mathrm{ij}}}{r_{j}^{*}},\frac{r_{\mathrm{ij}}}{r_{j}^{*}}\right),j\in B\;\left(\text{benefit criteria}\right),r_{j}^{*}=\max_{i}c_{\mathrm{ij}}\;\text{if}\;j\in B$$ (5)

$$A_{\mathrm{ij}}^{\mathrm{norm}}=\left(\frac{l_{j}^{-}}{r_{\mathrm{ij}}},\frac{l_{j}^{-}}{m_{\mathrm{ij}}},\frac{l_{j}^{-}}{l_{\mathrm{ij}}}\right),j\in C\;\left(\text{cost criteria}\right),l_{j}^{-}=\min_{i}c_{\mathrm{ij}}\;\text{if}\;j\in C$$ (6)

*B _ij_^norm^* of *Z _ij_* ^*norm*^ = *B _ij_* of *Z _ij_*

The order of Z-numbers, determined through the calculation of the degree of optimality, remains invariant. For example, if we have three alternatives expressed by Z-numbers, given by trapezoidal fuzzy numbers

$$Z_{1}=\left(6,7,8,9\right)\;\left(0.92,0.96,1,1\right)$$

$$Z_{2}=\left(4,5,6,7\right)\;\left(0.92,0.96,1,1\right)$$

$$Z_{3}=\left(8,9,10,10\right)\;\left(0.84,0.88,0.92,1\right)$$

Ranking these numbers, according to definition four, and calculating the appropriate degree of optimality, we have the following results:

*do*( *Z _1_,Z _2_*) = *1, do*( *Z _3_,Z _1_*) = *1, do*( *Z _2_,Z _1_*) = *0.17, do*( *Z1,Z2*) = *0.62,* and *Z _3_>Z _1_>Z _2_*

Normalizing Z-numbers, according to suggested approach, we obtain normalized values

$${Z_{1}}^{*}=\left(0.6,0.7,0.8,0.9\right)\;\left(0.92,0.96,1,1\right)$$

$${Z_{2}}^{*}=\left(0.4,0.5,0.6,0.7\right)\;\left(0.92,0.96,1,1\right)$$

$${Z_{3}}^{*}=\left(0.8,0.9,1,1\right)\;\left(0.84,0.88,0.92,1\right)$$

Then, after necessary calculations, we have

$$\mathrm{do}\left(Z_{1},Z_{2}\right)=1,\mathrm{do}\left(Z_{3},Z1\right)=1,\mathrm{do}\left(Z_{2},Z_{1}\right)=0.17,\mathrm{do}\left(Z_{1},Z_{2}\right)=0.62,\text{and}\;Z_{3}>Z_{1}>Z_{2}$$

Thus, approach provides relevant results and can be used.

Step 4. Constructing of the Z-number-based weighted normalized decision matrix.

Step 5. Defining the Z-number-based positive ideal solution and Z-number-based negative-ideal solution. In this paper, for Z-number based approach, as a positive-ideal solution we are using *Z _pis_* = (1,1), and as a negative-ideal solution *Z _nis_* = (0,0).

Step 6. Calculation of the distance from each alternative to the ideal-positive and ideal-negative solution.

Distance between two Z-numbers is calculated, as Z-number, based on definition four.

The distances of each *i*- *th* alternative from Z-number based positive-ideal solution (ZPIS) and Z-number based negative-ideal solution (ZNIS) are calculated as

$$d_{i}^{+}=\sum_{j=1}^{N}d\left(Z_{\mathrm{ij}},Z_{\mathrm{pis}}\right)$$ (7)

$$d_{i}^{-}=\sum_{j=1}^{N}d\left(Z_{\mathrm{ij}},Z_{\mathrm{nis}}\right)$$ (8)

here N – number of criteria.

Step 7. Calculation of the relative closeness to the best alternative

$$Z_{{\mathrm{cc}}_{i}}=\frac{d_{i}^{-}}{d_{i}^{+}+d_{i}^{-}}$$ (9)

Step 8. Ranking of the alternatives with the relative closeness.

***Z-PROMETHEE***

Classic and fuzzy PROMETHEE also has been widely applied for MCDM tasks solution. Let us briefly outline the features of Z-extension of this method based on direct calculations with Z-numbers.

Step 1 *.* Defining cost and benefit criteria.

Step 2. Construction of the initial decision matrix ( *ZDM _x_*) with *m* rows (alternatives) and *n* columns (criteria). Each element of the matrix is expressed by Z-number.

$${\mathrm{ZDM}}_{x}=\left[\begin{array}{cccc} z_{11} & z_{12} & \ldots & z_{1n}\\ z_{21} & z_{22} & \ldots & z_{2n}\\ \ldots & \ldots & & \ldots \\ z_{i1} & z_{i2} & \ldots & z_{\text{in}}\\ \ldots & \ldots & & \ldots \\ z_{m1} & z_{m2} & \ldots & z_{\mathrm{mn}} \end{array}\right]$$

Step 3.Normalization of the decision matrix

Normalization is performed in the same way as for Z-TOPSIS.

Step 4 *.* Defining Z-number based weights of criteria $Z_{{\mathrm{CW}}_{i}}$

Step 5 *.* Calculation of the differences between alternatives according to the degree

of dominance (definition three)

Step 6 *.* Calculation of the Z-values based preference function by using degree of optimality (definition three) according to the expressions

$$\begin{array}{c} P_{j}\left(Z_{\mathrm{ij}},Z_{\mathrm{ji}}\right)=0\;\text{if}\;\mathrm{do}\left(Z_{\mathrm{ij}},Z_{\mathrm{ji}}\right)\leq \mathrm{do}\left(Z_{\mathrm{ji}},Z_{\mathrm{ij}}\right)\\ P_{j}\left(Z_{\mathrm{ij}},Z_{\mathrm{ji}}\right)=\mathrm{do}\left(Z_{\mathrm{ij}},Z_{\mathrm{ji}}\right)-\mathrm{do}\left(Z_{\mathrm{ji}},Z_{\mathrm{ij}}\right)\;\text{if}\;\mathrm{do}\left(Z_{\mathrm{ij}},Z_{\mathrm{ji}}\right)\leq \mathrm{do}\left(Z_{\mathrm{ji}},Z_{\mathrm{ij}}\right) \end{array}$$ (10)

Step 7 *.* Calculation of the Z-number based weighted preference function

$$Z_{\mathrm{\pi w}}\left(Z_{\mathrm{ij}},Z_{\mathrm{ji}}\right)=P_{j}\left(Z_{\mathrm{ij}},Z_{\mathrm{ji}}\right)∙Z_{{\mathrm{CW}}_{i}}$$ (11)

Step 8 *.* Calculation of the leaving and entering flows for each alternative.

$$\Phi_{Z_{j}}^{+}\;\left(a\right)=\sum Z_{\mathrm{\pi w}}\left(a,b\right)$$ (12)

$$\Phi_{Z_{j}}^{-}\;\left(a\right)=\sum Z_{\mathrm{\pi w}}\left(b,a\right)$$ (13)

Step 9 *.* Net flows $\Phi_{Z_{j}}\;\left(a\right)$ are used for a complete ranking.

$$\Phi_{Z_{j}}\;\left(a\right)=\Phi_{Z_{j}}^{+}\;\left(a\right)-\Phi_{Z_{j}}^{-}\;\left(a\right)$$ (14)

***Calculation of importance weights***

Determination of criteria weights is one of the key stages in multi-criteria analysis. ^36^^–^^38^

In the case of expressing the weights of importance in Z-numbers, it should be taken into consideration that in comparison to crisp, fuzzy numbers or probabilistic values, Z-numbers reflect the opinion of experts in natural language and thus contain more complex information. Since most often weights are assigned based on an intuitive understanding of the relative importance of criteria, the use of Z-numbers allows expert to express his opinion in a way convenient for him. Moreover, Z-numbers enable the expression of the opinion of a group of experts more adequately. As a result, the general opinion of the experts should reflect a more relevant assessment of the criteria importance.

In this study, to reflect the linguistic expert’s assessment about weights more adequately, the usage of the Z-numbers-based swing method for setting the weights of the criteria is suggested.

*Swing method for Z-number based weights*

Expert assessments with Z-numbers allow to operate with more rich information. Weights can be assigned based on estimates of the criteria importance and confidence in these estimates. It is possible to evaluate the weights from lower level to higher. This approach looks like the defining of swing weights. ^39^ The key point is that the Z-number based weight of the more important criterion should dominate the weight of the less important criterion. This approach consists of several steps.

Step 1. A Z-number based swing weights matrix ( Table 1) is built, in which the upper part defines the importance of the criterion, and the left side represents the degree of confidence in it. A criterion that is very important for decision-making and there is great confidence in this, will be placed in the upper left corner of the matrix (cell labelled A). The criterion that has the least significance and the degree of confidence is placed in the lower right corner of the matrix (cell E).

**Table 1.** Swing weights matrix.

	Importance	Importance		
Confidence		High	Medium	Low
Confidence	High	A	B2	C3
	Medium	B1	C2	D2
	Low	C1	D1	E

Step 2. Consistency Rules. As in the case of the traditional swing weight matrix, the following rules are set to ensure consistency of the Z-number based weights.

If we accept *j* as cell label and Zwj as the non-normalized weight of importance indicated in the cells, then the following strict inequalities must be satisfied.

*Zw _A_>Zw _j_* for all other cells

$${\mathrm{Zw}}_{\text{B1}}>{\mathrm{Zw}}_{\text{C1}},{\mathrm{Zw}}_{\text{C2}},{\mathrm{Zw}}_{\text{D1}},{\mathrm{Zw}}_{\text{D2}},{\mathrm{Zw}}_{E},$$

$${\mathrm{Zw}}_{\text{B2}}>{\mathrm{Zw}}_{\text{C2}},{\mathrm{Zw}}_{\text{C3}},{\mathrm{Zw}}_{\text{D1}},{\mathrm{Zw}}_{\text{D2}},{\mathrm{Zw}}_{E},$$

$${\mathrm{Zw}}_{\text{C1}}>{\mathrm{Zw}}_{\text{D1}},{\mathrm{Zw}}_{E},$$

$${\mathrm{Zw}}_{\text{C2}}>{\mathrm{Zw}}_{\text{D1}},{\mathrm{Zw}}_{\text{D2}},{\mathrm{Zw}}_{E},$$

$${\mathrm{Zw}}_{\text{C3}}>{\mathrm{Zw}}_{\text{D2}},{\mathrm{Zw}}_{E},$$

$${\mathrm{Zw}}_{\text{D1}}>{\mathrm{Zw}}_{E}\;\text{and}{\mathrm{Zw}}_{\text{D2}}>{\mathrm{Zw}}_{E}$$

According to definition three, *Zw _B1_>Zw _B2_*, *Zw _C1_>Zw _C2_, Zw _C1_>Zw _C3_,Zw _D1_>Zw _D2_.* In case of asymmetrical fuzzy numbers results depends on degrees of optimality of *Zw _B1_*, *Zw _B2_*, *Zw _C1_ Zw _C2_,Zw _C3_*, *Zw _D1_*, *Zw _D2_*

Step 3. The criteria are placed in the cells of the matrix according to their importance values and the degree of confidence in them. Persons (experts, decision-makers, moderators) assigning weights to the Z-numbers must evaluate tradeoffs between level of importance and level of confidence. It should be noted that non-normalized importance weights are obtained at this stage. An example of swing Z-weight matrix is shown in Table 2.

**Table 2.** Example of Z-number based swing weights symmetric matrix.

	Level of importance			
Level of confidence	*C _2_ -* (VH,ES)	*C _3_, C _5_ -* (H,ES)	*C _6_ -* (A,ES)
	*C _4_ -* (VH,VS)	*C _1_ -* (H,VS)	
	*C _7_ -* (VH,S)		

Step 4. The assigned in the 3 ^rd^ step weights should be normalized according to below formula

$$Z_{{\text{Wnorm}}_{i}}=\frac{Z_{\mathrm{wi}}}{\sum_{i=1}^{\text{numberofcriteria}}Z_{w_{i}}}$$ (15)

In the example, the normalized weight of criteria *C _2_* is equal to

$$Z_{w_{\mathrm{norm}\;C2}}=\frac{\left(\mathrm{VH},\mathrm{ES}\right)}{\left(\mathrm{VH},\mathrm{ES}\right)+\left(\mathrm{VH},\mathrm{VS}\right)+2*\left(H,\mathrm{ES}\right)+\left(A,\mathrm{ES}\right)+\left(H,\mathrm{VS}\right)+\left(\mathrm{VH},S\right)}$$

### Criteria selection

As a starting point for identification of the location selection criteria, we have focused on the ideas related to investment, potential tourist preferences and geographical information of the territory. ^40^^–^^45^ We complemented this information with research findings from other publications on tourism facilities location selection. ^3^^–^^19^ Analysis shows that researchers have pointed out the economic and socio-cultural factors, features of natural resources and climatic, land size, transportation and infrastructure availability, accessibility, environment, land availability, labour availability, and legislation as the potential criteria. Variability of factors influencing the location selection depends on type and size of the tourist areas.

To identify the criteria, a group of nine experts from the tourism sector, state agency and universities was formed. The preliminary list of nine potential experts was composed based on subject area and general competencies of the specialists. Given that the research topics directly related to the tourism development in Azerbaijan, all potential experts were selected from Azerbaijan. At the first stage, the experts analysed prepared criteria list and selected six criteria. After that, five experts confirmed readiness to participate in the next round. The group of five experts was composed for evaluation of the selected criteria. Before opinion studies, information on linguistic terms and presentation of these terms as Z-numbers was sent to the experts. Each expert provided evaluations of the criteria. We organised expert group meetings for deriving consensus-based evaluation of the criteria. Each expert had opportunity to get information on other expert’s opinions and discuss it. After discussions and justifications of opinions, a consensus based final opinion was outlined by the expert group. For the questionnaire given to experts in the discussion meetings, see *Extended data.* ^47^

## Results

### Tourism location sites selection in the Republic of Azerbaijan

The Republic of Azerbaijan is divided into 14 economic regions. Each of these regions has a specificity of economic development and a certain potential for tourism development. The nine climatic zones, as well as a variety of the available recreational resources, determine the role of tourism as a driver of sustainable development in areas with tourism and recreational services development potentials.

**Figure 1.**
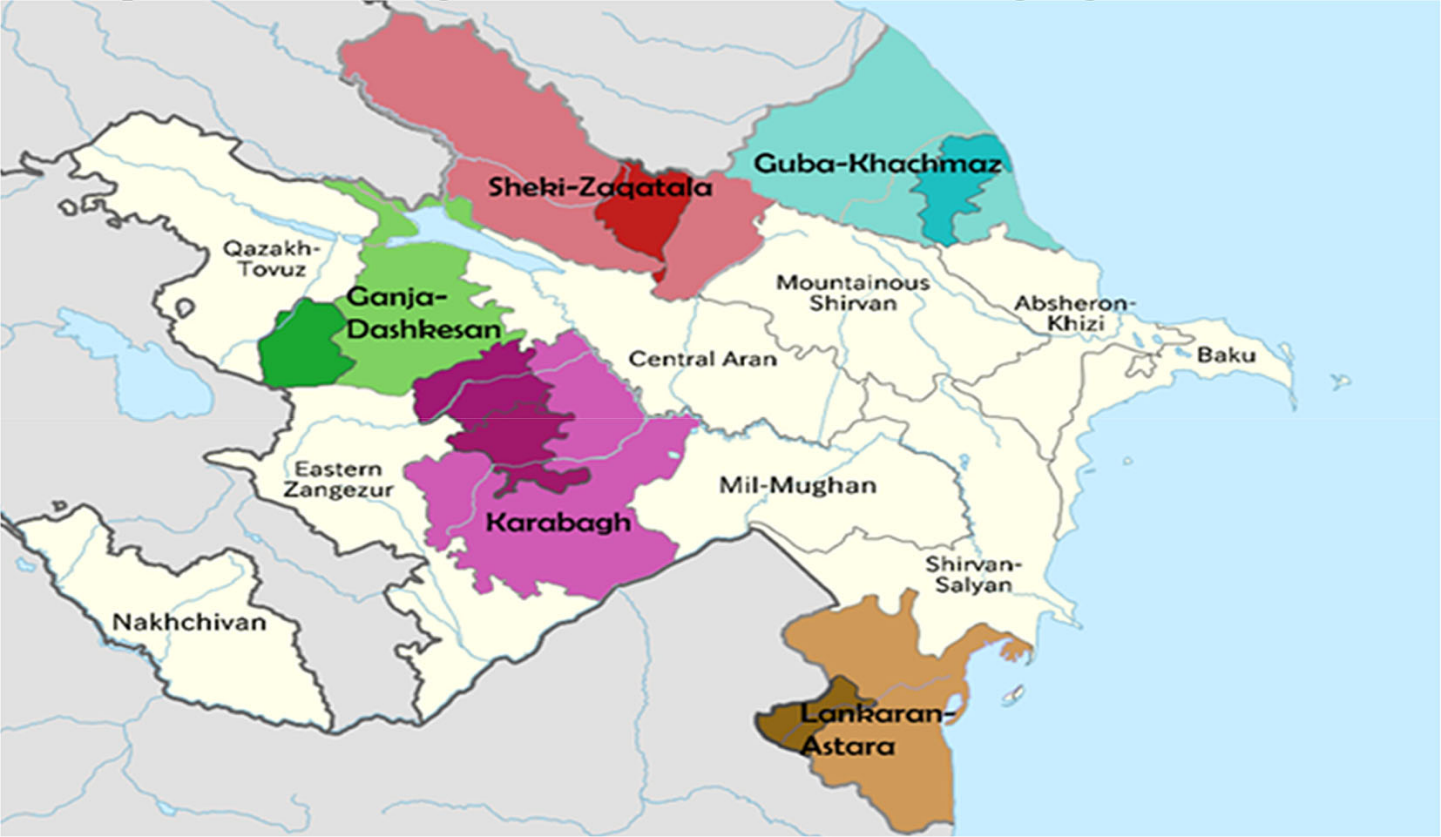
Map of Azerbaijan Republic with studied regions (Source: Wikimedia).

Five regions of the country (Quba-Khachmaz, Lankaran-Astara, Shaki-Zagatala, Ganja-Dashkasan and Karabakh) are considered as the attractive areas for tourism development. ^46^ Figure 1 represents a map on which the regions under consideration are highlighted.

The results of the study have to rank potential regions/districts for tourism development that can contribute to the development of tourism in the country and sustainable development of the region.

Our research is aimed at finding tourism development site selection at the meta-level - region/district. After the successful tourism location selection at the meta-level, further study and calculations should be continued.

**Criteria for tourism development locations selection**

For the case of Azerbaijan, the following criteria were selected:

• Economical criterion
• Recreation and tourism resource availability
• Accessibility
• Ecological and environmental attractiveness
• Infrastructure and utilities availability
• Human resources

The criteria are as follows. The *Economical* criterion implies an integral assessment of the volume of the necessary investments, which at this stage of decision-making are expressed by linguistic values - "large", "significant", "average" based on the cognitive ideas of experts. Each expert has its own semantics of these values, but there are some general ideas. For example, if volume of investments is 50 million manats ($29 million US dollars), then almost all experts will agree that the linguistic value of this criterion will be “high” or “very high”. The criterion *Recreation and tourism resources* is an integral assessment of availability and conditions of natural areas, forest, mineral waters etc. and man-made resources. *Accessibility* criterion is related to the presence and situation with roads and railways, air traffic, as well as the ability to access this place regardless of natural conditions. *Ecological and environmental attractiveness* implies considering the ecological possibilities of the region. In our opinion, taking this criterion into account is an important approach to ensure sustainable development of the selected tourism area. At present, a combination of unique naturalness and attractiveness is highly demanded, and this indicator has an integral character. An ecologically unfavourable territory may have a unique landscape, or vice versa - an ecologically clean territory may not have attractive natural objects. Although, such places can be attractive for some travellers, for site locations such places are not used. *Utilities availabilities* provide an integrated assessment about water, electricity, and gas in given region. These components play a key role in economic and social life of the region, and high level of utilities is pivotal precondition of further sustainable development. *Human resources* include availability of the necessary labour force for development activity and for site maintenance.

### Z-number based normalized decision matrix and criteria weighting

*Z-evaluation of alternatives and construction of the decision matrix*

The five experts evaluated each criterion for all alternatives using Z-numbers. The result of evaluation is shown in Table 3. ^47^

**Table 3.** Z-number based value of criteria for all alternatives.

Expert	Alternative	Environmental and ecological attractiveness C1	Recreation and tourism resource C2	Economic criteria C3	Accessibility C4	Utilities C5	Human resources C6
*E _1_*	S-Z	VH,ES	H,ES	A,ES	H,VS	A, VS	H,S
	L-A	H,VS	A,VS	H,ES	A,S	A,S	A,S
	G-D	H,VS	H,VS	VH,VS	H,S	A,VS	A,VS
	Q-K	H,ES	A,VS	L,VS	VH,VS	H,S	H,S
	K	VH,VS	H,VS	A,S	H,VS	A,S	VH,VS
*E _2_*	S-Z	VH,ES	H,VS	A,VS	H,VS	H,S	H,S
	L-A	H,S	A,VS	H,ES	A,VS	A,VS	A,VS
	G-D	VH,VS	H,VS	VH,VS	A,VS	A,VS	A,VS
	Q-K	H,VS	A,VS	L,ES	VH,VS	H,S	H,S
	K	VH,VS	H,ES	A,VS	H,VS	A,S	H,VS
*E _3_*	S-Z	H,ES	H,VS	H,ES	H,VS	A,VS	VH,S
	L-A	H,VS	H,S	H,VS	A,VS	A,S	A,S
	G-D	VH,VS	H,S	H,VS	A,VS	H,S	A,VS
	Q-K	VH,S	H,VS	A,VS	VH,ES	H,VS	H,S
	K	VH,ES	H,VS	A,ES	H,VS	A,VS	H,ES
*E _4_*	S-Z	VH,VS	H,VS	A,VS	H,VS	A,VS	H,VS
	L-A	A,VS	A,S	H,VS	A,S	A,S	A,VS
	G-D	H,ES	H,ES	H,ES	H,S	A,VS	A,S
	Q-K	H,VS	H,ES	A, VS	VH,VS	H,VS	H,VS
	K	VH,S	H,VS	H,ES	VH,VS	A,S	H,ES
*E _5_*	S-Z	H,VS	H,ES	A,VS	H,VS	H,VS	H,S
	L-A	H,VS	A,S	H,VS	A,VS	A,S	A,VS
	G-D	VH,ES	H,VS	H,ES	H,S	A,VS	A,VS
	Q-K	H,S	H,ES	A,S	VH,ES	H,VS	A,S
	K	VH,ES	H,ES	A,S	H,VS	A,VS	H,S

Here S-Z – Shaki-Zagatala, L-S – Lankaran-Astara, G-D- Ganja-Dashakasan, Q-K - Quba-Khachmaz, K- Karabakh regions.

The linguistic values are expressed by the following fuzzy numbers with trapezoidal membership functions:

VH – *Very High*- (8,9,10,10);

H- *High* – (6,7,8,9);

A *- Average* – (4,5,6,7);

ES - *Extremely Sure* – (0.92,0.96, 1, 1);

VS – *Very Sure* – (0.84, 0.88,0.92, 0.96);

S - *Sure –* (0.76, 0.8,0.84,0.88).

Moderator also estimated the competence of experts using Z-numbers. The Z-estimations provided by Moderator are presented in Table 4.

**Table 4.** Z-number based expert’s competence estimates.

Expert	Z-estimation of competencies	Parts A and B of Z-estimation, expressed by trapezoidal FN	Weighted Z-value of competence
1	VH, ES	(8 9 10 10) (0.92 0.96 1 1)	(0.167 0.196 0.244 0.278) (0.756 0.838 0.92 0.96)
2	VH, VS	(8 9 10 10) (0.84 0.88 0.92 0.96)	(0.167 0.196 0.244 0.278) (0.67 0.753 0.837 0.919)
3	H, ES	(6 7 8 9) (0.92 0.96 1 1)	(0.125 0.152 0.195 0.25) (0.705 0.789 0.882 0.94)
4	H, VS	(6 7 8 9) (0.84 0.88 0.92 0.96)	(0.125 0.152 0.195 0.25) (0.643 0.723 0.811 0.902)
5	VH, VS	(8 9 10 10) (0.84 0.88 0.92 0.96)	(0.167 0.196 0.244 0.278) (0.67 0.753 0.837 0.919)

Here linguistic values are expressed by the following fuzzy numbers with trapezoidal membership functions:

VH – *very high* with trapezoidal MF - (8, 9, 10, 10);

H – *high*- (6, 7, 8, 9);

ES – *extremely sure*- (0.92, 0.96, 1, 1);

VS – *very sure*- (0.84, 0.88, 0.92, 0.96).

Weighted Z-value of competence of *i*-th expert is calculated by the following formula

$$Z_{{\text{Wexp}}_{i}}=\frac{Z_{\text{esti}}}{\sum_{i=1}^{N}Z_{\text{esti}}}$$ (16)

here *Zest _i_ -* Z-estimation of competence of *i ^th^* expert, N- number of experts

Why are different assessments of competence obtained? The moderator can know the expert very well and be completely confident in their competence. There may also be a situation when the invited person has all signs of a very good specialist, but the moderator is less confident in the expert's competence, in the field of the problem being under consideration.

For construction of the Z-numbers based decision matrix the appropriate values from Table 3 multiplied by the competence weights of experts from Table 4. Then decision matrix is normalized. The results are shown in Table 5. The alternatives are denoted as: A1 (Shaki-Zagatala), A2 (Lankaran-Astara), A3 (Ganja-Dashkasan), A4 (Quba-Khachmaz) and A5 (Karabakh).

**Table 5.** Z-number based normalized decision matrix.

Criteria	Alternative	Part A				Part B			
C1	A1	0.282559	0.426532	0.629899	0.921751	0.097	0.182	0.309	0.592
	A2	0.212256	0.33468	0.510303	0.765253	0.059	0.119	0.206	0.45
	A3	0.282559	0.426532	0.629899	0.921751	0.063	0.139	0.243	0.519
	A4	0.241616	0.374007	0.562559	0.834747	0.056	0.118	0.207	0.445
	A5	0.31771	0.472458	0.689697	1	0.066	0.133	0.234	0.507
C2	A1	0.28367	0.442929	0.670539	1	0.07	0.145	0.254	0.54
	A2	0.188552	0.319865	0.511616	0.793434	0.05	0.101	0.179	0.392
	A3	0.28367	0.442929	0.670539	1	0.062	0.125	0.22	0.476
	A4	0.232492	0.377273	0.586364	0.891246	0.071	0.143	0.251	0.541
	A5	0.28367	0.442929	0.670539	1	0.07	0.14	0.253	0.539
C3	A1	0.150011	0.232642	0.372105	0.63125	0.07	0.145	0.254	0.54
	A2	0.119024	0.177504	0.268719	0.419585	0.068	0.143	0.254	0.536
	A3	0.107349	0.157707	0.234028	0.355455	0.097	0.179	0.299	0.588
	A4	0.185613	0.301493	0.518328	1	0.06	0.122	0.221	0.474
	A5	0.150011	0.232642	0.372105	0.63125	0.063	0.126	0.216	0.476
C4	A1	0.226936	0.354343	0.536431	0.8	0.062	0.125	0.221	0.478
	A2	0.136162	0.236229	0.383165	0.6	0.054	0.111	0.19	0.42
	A3	0.191785	0.308418	0.476633	0.721751	0.045	0.099	0.174	0.377
	A4	0.31771	0.472458	0.689697	1	0.071	0.143	0.251	0.541
	A5	0.241616	0.374007	0.562559	0.834747	0.062	0.125	0.221	0.478
C5	A1	0.22138	0.360943	0.563131	0.858754	0.052	0.114	0.2	0.43
	A2	0.170202	0.295286	0.478956	0.75	0.047	0.096	0.165	0.368
	A3	0.188552	0.319865	0.511616	0.793434	0.057	0.117	0.206	0.448
	A4	0.28367	0.442929	0.670539	1	0.056	0.113	0.188	0.421
	A5	0.170202	0.295286	0.478956	0.75	0.052	0.105	0.176	0.394
C6	A1	0.286444	0.443398	0.666933	0.989622	0.048	0.098	0.164	0.369
	A2	0.161424	0.280057	0.454255	0.71132	0.054	0.111	0.19	0.42
	A3	0.161424	0.280057	0.454255	0.71132	0.057	0.117	0.206	0.448
	A4	0.244771	0.388951	0.59604	0.896855	0.048	0.098	0.164	0.369
	A5	0.29331	0.451221	0.675874	1	0.054	0.121	0.212	0.455

*Weights of criteria*

After discussion, the expert group constructed consensus-based swing Z-weight matrix ( Table 6).

**Table 6.** Z-number based swing weights matrix.

		Importance		
		High	Medium	Low
Confidence	High	C1- VI, ES	C3 - I, ES	
	Medium	C2 - VI, VS	C4, C5- I,VS	
	Low			C6 - I,S

The weights of criteria, expressed by Z-numbers, are calculated according to the formula (15). The results are shown in Table 7.

**Table 7.** Z-number based weights of criteria.

Criteria	Part A	Part B
Environmental and ecological attractiveness	0.148 0.18 0.227 0.263	0.704 0.785 0.854 0.909
Recreation and tourism resource	0.148 0.18 0.227 0.263	0.647 0.726 0.805 0.881
Economic criteria	0.111 0.14 0.182 0.237	0.66 0.737 0.812 0.88
Accessibility	0.111 0.14 0.182 0.237	0.619 0.692 0.771 0.854
Utilities availability	0.111 0.14 0.182 0.237	0.619 0.692 0.771 0.854
Human Resources	0.074 0.1 0.136 0.184	0.708 0.771 0.827 0.874

*Z-TOPSIS based solution*

After compilation of the normalized decision matrix and table of criteria weights, the following TOPSIS-specific calculations are performed.

Step 1. Constructing of the Z-number-based weighted normalized decision matrix ( Table 8).

**Table 8.** Z-number-based weighted normalized decision matrix.

Cri-teria	Alternative	Part A				Part B			
C1	A1	0.042	0.077	0.143	0.242	0.089	0.172	0.292	0.556
	A2	0.031	0.060	0.116	0.201	0.059	0.119	0.206	0.450
	A3	0.042	0.077	0.143	0.242	0.059	0.136	0.234	0.488
	A4	0.036	0.067	0.128	0.220	0.039	0.093	0.187	0.405
	A5	0.047	0.085	0.157	0.263	0.049	0.114	0.217	0.491
C2	A1	0.042	0.080	0.152	0.263	0.045	0.105	0.204	0.476
	A2	0.028	0.058	0.116	0.209	0.032	0.073	0.144	0.345
	A3	0.042	0.080	0.152	0.263	0.040	0.091	0.177	0.419
	A4	0.034	0.068	0.133	0.234	0.046	0.104	0.202	0.477
	A5	0.042	0.080	0.152	0.263	0.045	0.102	0.204	0.475
C3	A1	0.017	0.033	0.068	0.150	0.046	0.107	0.206	0.475
	A2	0.013	0.025	0.049	0.099	0.045	0.105	0.206	0.472
	A3	0.012	0.022	0.043	0.084	0.064	0.132	0.243	0.517
	A4	0.021	0.042	0.094	0.237	0.040	0.090	0.179	0.417
	A5	0.017	0.033	0.068	0.150	0.042	0.093	0.175	0.419
C4	A1	0.025	0.050	0.098	0.190	0.038	0.087	0.170	0.408
	A2	0.015	0.033	0.070	0.142	0.033	0.077	0.146	0.359
	A3	0.021	0.043	0.087	0.171	0.028	0.069	0.134	0.322
	A4	0.035	0.066	0.126	0.237	0.044	0.099	0.194	0.462
	A5	0.027	0.052	0.102	0.198	0.038	0.087	0.170	0.408
C5	A1	0.025	0.051	0.102	0.204	0.032	0.079	0.154	0.367
	A2	0.019	0.041	0.087	0.178	0.029	0.066	0.127	0.314
	A3	0.021	0.045	0.093	0.188	0.035	0.081	0.159	0.383
	A4	0.031	0.062	0.122	0.237	0.035	0.078	0.155	0.360
	A5	0.019	0.041	0.087	0.178	0.032	0.073	0.136	0.336
C6	A1	0.021	0.044	0.091	0.182	0.034	0.076	0.136	0.323
	A2	0.012	0.028	0.062	0.131	0.038	0.086	0.157	0.367
	A3	0.012	0.028	0.062	0.131	0.040	0.090	0.170	0.392
	A4	0.018	0.039	0.081	0.165	0.034	0.076	0.146	0.323
	A5	0.022	0.045	0.092	0.184	0.038	0.093	0.175	0.398

Step 2. Calculation of the distance from each alternative to the ideal-positive and ideal-negative solution and calculation of the relative closeness to the best alternative.

Distances between two Z-numbers and Z-number based closeness coefficients are calculated in accordance with the definition four and formula (9). The results are presented in the Table 9.

**Table 9.** Distance and closeness of alternatives.

Alternative	Distance between Z-PIS	Distance between Z-NIS	Closeness
A1	20.467	3.533	0.147
A2	21.059	2.941	0.123
A3	20.696	3.304	0.138
A4	20.592	3.408	0.142
A5	20.594	3.406	0.142

Step 3. Ranking of alternatives with the relative closeness

By ranking the alternatives in accordance with the closeness we obtain the following order of the alternatives: A1, A4, A5, A3, and A2. Higher priority has alternative A.

### Z-PROMETHEE based solution

After composition of the decision matrix, finding of the best alternative is realized as a multi-step process.

Step 1. Determination of the differences between alternatives and calculation the Z-values based preference function according to the expression 10.

At this step, Z-number based values of the criteria for each alternative are compared in pairs according to the optimality degree (definition three). Then, based on the optimality degree, the preference function ( formula 10) is calculated.

Step 2. Z-number based weighted preferences ( formula 11) are obtained by multiplying the appropriate values of preference function and Z-number based weights of criteria from Table 10. As a result, we get the following table of weighted preferences ( Table 10)

**Table 10.** Z-number based weighted preference function.

Alternatives	Part A				Part B			
A1-A2	0.182	0.226	0.291	0.359	0.163	0.238	0.353	0.520
A1-A3	0.089	0.113	0.145	0.185	0.252	0.319	0.436	0.603
A1-A4	0.054	0.067	0.085	0.100	0.387	0.489	0.628	0.755
A1-A5	0.038	0.047	0.061	0.077	0.380	0.456	0.600	0.740
A2-A1	0.000	0.000	0.000	0.000	0.082	0.155	0.274	0.449
A2-A3	0.000	0.000	0.000	0.000	0.082	0.155	0.274	0.449
A2-A4	0.000	0.000	0.000	0.000	0.082	0.155	0.274	0.449
A2-A5	0.000	0.000	0.000	0.000	0.082	0.155	0.274	0.449
A3-A1	0.000	0.000	0.000	0.000	0.082	0.155	0.274	0.449
A3-A2	0.099	0.121	0.154	0.186	0.191	0.216	0.238	0.257
A3-A4	0.022	0.027	0.034	0.039	0.082	0.155	0.274	0.449
A3-A5	0.011	0.014	0.018	0.024	0.082	0.155	0.274	0.449
A4-A1	0.049	0.062	0.080	0.104	0.497	0.583	0.680	0.789
A4-A2	0.176	0.221	0.285	0.363	0.371	0.466	0.578	0.680
A4-A3	0.105	0.133	0.174	0.226	0.130	0.156	0.185	0.218
A4-A5	0.072	0.091	0.117	0.153	0.447	0.536	0.640	0.746
A5-A1	0.010	0.013	0.018	0.024	0.082	0.155	0.274	0.449
A5-A2	0.161	0.201	0.260	0.321	0.127	0.212	0.333	0.498
A5-A3	0.077	0.098	0.127	0.165	0.278	0.282	0.285	0.287
A5-A4	0.056	0.070	0.091	0.111	0.403	0.419	0.437	0.458

Step 3. Calculation of the leaving and entering flows for each alternative. The results of the calculation of the net flows are shown in Table 11.

**Table 11.** Z-number based net flows.

Alternatives	Z-value of net flow	
	Part A	Part B
A1	0.2350.3550.5070.662	0.00000010.00000290.00006620.0010787
A2	-1.206-0.96-0.73-0.568	0.00058680.00230070.00826310.0271535
A3	-0.34-0.1390.0540.249	0.00017370.00048080.00078930.0027116
A4	0.1520.2970.4920.714	0.00008040.00038130.00178180.0052415
A5	0.040.1730.3260.476	0.00001610.00014720.00119940.0072920

Step 4. Ranking of the alternatives with the Z-number based values of net flows.

By ranking the alternatives in accordance with the dominance value, calculated according to definition three, we obtain the following priority order for alternatives: A1, A4, A5, A3, and A2.

## Discussion

Analysis of the literature shows that for decision-making in complicated cases, two or more techniques are often utilized. As a final decision, the solution ranked as the best option by several methods is selected. In case of discrepancy, additional studies are carried out to obtain an adequate solution.

In this paper, two MCDM models based on the use of Z-numbers were developed for tourism development site selection; Z-TOPSIS and Z-PROMETHEE. Models were developed based on subject area experts and decision-makers knowledge. Heterogeneity and complexity of tourism sector, as well as specificity of task, necessitate the use of Z-numbers as formalism for describing high level uncertainty. Z-numbers allow experts to assess factors affecting location selection in terms of natural language.

Z-numbers were used for criteria weighting and evaluations, for estimation of the expert’s competences, for the decision matrix construction, as well as, in implementation of the direct calculation techniques with Z-numbers for the problem solution. Implementation of the direct calculations techniques with Z-numbers allows to solve decision-making task without transformations that distort the original information and, as a result, affect the final decision.

Moreover, a new approach for normalization of the Z-number-based decision matrix was proposed. This approach allows to expand TOPSIS and PROMETHEE methods for direct use of the Z-numbers. The solution for the tourism site selection task by both methods provided the same result in determining the best alternative and alternatives ranking. For the determination of the criteria weights, the swing weighting method was extended for operating with Z-numbers.

Six important criteria are used for decision-making on selecting suitable regions for tourism development. Results of the research enabled the identification of three regions of Azerbaijan that are most suitable for the tourism development, namely, Sheki-Zagatala, Guba-Khachmaz and Karabakh economic regions. The study showed the importance of the such nature-based criteria, as *environmental/ecological attractiveness* and *recreation and tourism resource*, in decision-making aimed at sustainable development of regions through the extension of the tourism sphere.

## Conclusions

Z-numbers formalism has been successfully used for development of the MCDM models for tourism development site selection in case of imperfect information and high-level uncertainty. For the problem solution, outranking and far from ideal solution techniques are used.

Research has shown the effectiveness of Z-numbers for imperfect information formalization, and reliable decision-making in conditions of high-level uncertainty by applying Z-extensions of the various MCDM methods. The results obtained make it possible to use the formalism of Z-numbers not only for description, but also for carrying out the necessary calculations, considering the degree of reliability of the available initial information.

The successful application of the Z-number based swing matrix for determining the criteria importance weights, and suggested approach for normalization of the Z-number-based decision matrix allows for the use of these tools as the weighting and normalization technique for other multi-criteria methods dealing with imperfect information.

Direct calculations with Z-numbers, and application of the Z-calc software for solution of the Z-numbers based MCDM task for tourism development site selection, showed the importance of having appropriate software. For further research with Z-numbers based models and methods, it is important to extend computational procedures in accordance with requirements of various MCDM and to pay considerable care to the availability of appropriate software, to support the multi-criteria approaches. The creation of user-friendly web-software will make it possible to take advantage of the Z-numbers more widely for managerial decision-making in various areas.

## Data availability

### Underlying data

**Figshare:** Application of the expert knowledge-based fuzzy MCDM models for selection of the tourism development site: the case of Azerbaijan

https://doi.org/10.6084/m9.figshare.c.5871563.v3 ^47^

The project contains the following underlying data:

• Preference-function.xlsx
• Z-number-based-Decision-Matrix-initial-non-normalized.xlsx.
• Evaluation of alternatives and decision matrix.docx
• Criteria importance evaluation.docx

### Extended data

**Figshare:** Application of the expert knowledge-based fuzzy MCDM models for selection of the tourism development site: the case of Azerbaijan

https://doi.org/10.6084/m9.figshare.c.5871563.v3 ^47^

The project contains the following extended data:

• Delphi-first round questionnaire.docx

Data are available under the terms of the Creative Commons Attribution 4.0 International license (CC-BY 4.0).

## F1000 research statement of endorsement

Professor Latafat Gardashova confirms that the author has an appropriate level of expertise to conduct this research and confirms that the submission is of an acceptable scientific standard. Professor Latafat Gardashova declares they have no competing interests. Affiliation: Azerbaijan State Oil and Industry University.
