# Supplementary material for: Fuzzy MCDM models for selection of the tourism development site: the case of Azerbaijan
Source: F1000Res. 2022 Mar 14;11:310. [Version 1] doi: 10.12688/f1000research.109709.1 (PMC8931446; doi:10.12688/f1000research.109709.1)
Supplement: Peer review history

# Peer review: Reviewer response for version 1

**DOI:** 10.5256/f1000research.121249.r127456

**Published:** 2022-03-28

**Authors:** Rustam Vahidov

**Affiliations:** 1 Concordia University, Montreal, QC, Canada

The article proposes a method based on so called Z numbers for tackling tourist site selection problem as applied to the country of Azerbaijan. The notion of Z numbers is introduced as being composed of a pair of fuzzy numbers, first one indicating the value, and the second one the certainty of the value. Relevant operations are defined. A thorough review of the MCDM method is provided. The article enhances the TOPSIS and PROMETHEE methods using Z numbers. The application of these new enhanced methods to tourist site selection is provided. The article makes a clear contribution to the literature by providing selection methods in cases where statistical information is lacking.

Is the work clearly and accurately presented and does it cite the current literature?

Yes

If applicable, is the statistical analysis and its interpretation appropriate?

Not applicable

Are all the source data underlying the results available to ensure full reproducibility?

Yes

Is the study design appropriate and is the work technically sound?

Yes

Are the conclusions drawn adequately supported by the results?

Yes

Are sufficient details of methods and analysis provided to allow replication by others?

Yes

Reviewer Expertise:

Decision Support, Electronic Negotiations, Application of Fuzzy sets and systems, neural networks, evolutionary algorithms

I confirm that I have read this submission and believe that I have an appropriate level of expertise to confirm that it is of an acceptable scientific standard.

# Peer review: Reviewer response for version 1

**DOI:** 10.5256/f1000research.121249.r127452

**Published:** 2022-03-17

**Authors:** Babek Guirimov

**Affiliations:** 1 State Oil Company of Azerbaijan Republic (SOCAR), Baku, Azerbaijan

The paper suggests an approach based on the Z-numbers based extension of TOPSIS and PROMETHEE methods for the selection of a tourism development site. Preliminary information to introduce the reader to Z-number theory, including arithmetical operations and ranking is provided. The details of Z-TOPSIS and Z-PROMETHEE implementation principles are described with examples. A specific example is used to demonstrate the applicability of the method. The results demonstrated that the suggested approach can be successfully used for the development of multi-criteria decision making models, particularly in the area of the tourism industry. The paper is interesting and can be indexed.

Is the work clearly and accurately presented and does it cite the current literature?

Yes

If applicable, is the statistical analysis and its interpretation appropriate?

Yes

Are all the source data underlying the results available to ensure full reproducibility?

Yes

Is the study design appropriate and is the work technically sound?

Yes

Are the conclusions drawn adequately supported by the results?

Yes

Are sufficient details of methods and analysis provided to allow replication by others?

Yes

Reviewer Expertise:

Soft Computing, Computational Intelligence

I confirm that I have read this submission and believe that I have an appropriate level of expertise to confirm that it is of an acceptable scientific standard.

# Peer review: Reviewer response for version 1

**DOI:** 10.5256/f1000research.121249.r127453

**Published:** 2022-03-17

**Authors:** Mehmet Ali Salahli

**Affiliations:** 1 Educational Technologies Application and Research Center, Çanakkale Onsekiz Mart University, Çanakkale, Turkey

Scientifically well-defined selection of tourism sites is an important factor in the development of the tourism industry. This problem is very important especially for countries whose tourism industry is not very developed. In this sense, the subject of the study is current.

 The aim of the study is well expressed. In particular, the concepts that form the scientific basis of the study and the method of the study are adequately explained by referring to the sources.

 There is not enough statistical data for the application area of ​​the study and the reliability of the available data is low. In such a case, it is correct to use the MCDM method based to Z-Numbers.

 Criteria for the solution of the problem have been chosen correctly.

 The structure of the research is good. In particular, a comprehensive list of resources on Z fuzzy and MMKS is provided.

 I think that the study will be useful in finding effective solutions in the field of application and similar areas.

Is the work clearly and accurately presented and does it cite the current literature?

Yes

If applicable, is the statistical analysis and its interpretation appropriate?

Not applicable

Are all the source data underlying the results available to ensure full reproducibility?

Yes

Is the study design appropriate and is the work technically sound?

Yes

Are the conclusions drawn adequately supported by the results?

Yes

Are sufficient details of methods and analysis provided to allow replication by others?

Yes

Reviewer Expertise:

fuzzy applications, data mining, knowledge discovery

I confirm that I have read this submission and believe that I have an appropriate level of expertise to confirm that it is of an acceptable scientific standard.
